# RETRACTION: Dose‐ and Time‐Dependent Apoptosis Induced by Avian H9N2 Influenza Virus in Human Cells

**DOI:** 10.1155/bmri/9831907

**Published:** 2025-10-18

**Authors:** BioMed Research International

RETRACTION: S. Shahsavandi, M.M. Ebrahimi, K. Sadeghi, S.Z. Mosavi, and A. Mohammadi, “Dose‐ and Time‐Dependent Apoptosis Induced by Avian H9N2 Influenza Virus in Human Cells,” *BioMed Research International* 2013 (2013): 524165, https://doi.org/10.1155/2013/524165.

The above article, published online on 11 September 2013 in Wiley Online Library (wileyonlinelibrary.com), has been retracted following the agreement of the journal’s Section Editor, Jinsong Ren; and John Wiley & Sons Ltd.

The retraction has been agreed following an investigation of the concerns raised by *Penicillium citreoviride* and *Actinopolyspora biskrensis* on PubPeer [1], which identified several concerns related to Figure 3 where every panel displays unexpectedly overlapping features.

In addition, concerns have been raised regarding the similarity of the Mock HepG2 cells shown in Figure 1 to the Mock HLM cells in Figure 1 in another publication [2].

As a result of the investigation, the data and conclusions of this article are considered unreliable.

The authors disagree with this retraction.
